# Recall-by-genotype of neurodevelopmental disorder copy number variants in a multi-ancestry, healthcare-system biobank

**DOI:** 10.1038/s41525-026-00597-6

**Published:** 2026-07-16

**Authors:** Nina Zaks, Behrang Mahjani, Abraham Reichenberg, Rebecca Birnbaum

**Affiliations:** 1 Department of Child and Adolescent Psychiatry, NYU Langone Health, New York, NY USA; 2 Department of Psychiatry, Icahn School of Medicine at Mount Sinai, New York, NY USA; 3 Seaver Autism Center for Research and Treatment, Icahn School of Medicine at Mount Sinai, New York, NY USA; 4 Department of Environmental Medicine and Public Health, Icahn School of Medicine at Mount Sinai, New York, NY USA; 5 Department of Genetics and Genomics Sciences, Icahn School of Medicine at Mount Sinai, New York, NY USA

**Keywords:** Diseases, Genetics, Medical research, Neuroscience

## Abstract

Clinical biobanks linking electronic health records (EHRs) with genotype data enable the study of genomic risk factors in real-world populations. However, recall-by-genotype (RbG) of psychiatric risk variants in diverse healthcare-system biobanks remains scarce. Leveraging Bio*Me*, a multi-ancestry biobank within the Mount Sinai Health System, we recalled carriers of rare copy number variants (CNVs) that confer increased risk for neurodevelopmental disorders (NDDs) to establish empirical benchmarks for RbG implementation. We recontacted 892 participants: 335 NDD CNV carriers, 217 individuals with schizophrenia without NDD CNVs, and 340 neurotypical controls without NDD CNVs. Participants completed clinical and cognitive assessments. Overall, 18% of recontacted participants responded to recruitment, and 8% completed the study: 30 NDD CNV carriers, 20 individuals with schizophrenia, and 23 controls. The mean age was 48.8 years, 66% were female, and self-reported ancestry was 37% African, 34% Hispanic, and 26% European. Seventy percent of NDD CNV carriers had at least one neuropsychiatric or developmental condition, including mood or anxiety disorders (40%). Among 22 NDD CNV carriers at loci implicated in impaired cognition, performance was lower than controls on Digit Span Backward (*β* = –1.76, FDR = 0.04) and Digit Span Sequencing (*β* = −2.01, FDR = 0.04). NDD CNV carriers also outperformed the schizophrenia group on verbal learning (*β* = 4.5, FDR = 0.05). Recall of individuals—including those with psychiatric illness—yielded phenotypes not captured in EHRs and provides empirical benchmarks relevant to RbG implementation and precision psychiatry in diverse healthcare systems.

## Introduction

Clinical biobanks linking electronic health records (EHRs) with genomic data are rapidly expanding, creating new opportunities to investigate disease-relevant genomic variation in real-world, diverse populations^1–4^. However, compared with numerous in silico and computational analyses of biobank data, relatively few recall-by-genotype (RbG) studies have been published, especially those focused on psychiatric risk variants in diverse healthcare-system biobanks^5–11^. RbG is conceptually related to reverse phenotyping and genotype-first approaches but is distinguished by prospective, systematic recontact of individuals identified through genomic data, with standardized phenotyping^9,12^. RbG enables recruitment of carriers of specific genomic variants for deep phenotyping and observational or interventional studies, complementing EHR-based analyses. Empirical data on RbG implementation and phenotypic yield—particularly in psychiatric populations—are needed to inform study design, scalability, and interpretation.

Some rare, recurrent copy number variants (CNVs)—genomic microdeletions or microduplications typically spanning tens of kilobases to several megabases—confer markedly increased risk for multiple neurodevelopmental disorders (NDDs) and are among the highest-effect genetic risk factors in neuropsychiatry^13–18^. As such, identifying NDD CNV carriers may facilitate elucidation of genotype–phenotype relationships and inform future RbG studies and characterization of clinical outcomes. Collectively, NDD CNVs are observed in a substantial proportion of individuals with NDDs, including up to 20% of autism cases, ~14% of developmental delay and intellectual disability, and 1–3% of schizophrenia cases^18–23^. NDD CNVs also negatively affect neurocognition, a quantitative trait linked to functional outcomes, as reported in clinically ascertained cohorts^19,24^. Prior studies in population samples—including the Icelandic population cohort and UK Biobank—demonstrated cognitive effects even among neurotypical individuals without psychiatric illness^24–28^. NDD CNVs exhibit variable penetrance and expressivity, resulting in heterogeneous outcomes ranging from unaffected to severely affected^29–31^. Although individual NDD CNVs are rare, their combined prevalence has been estimated at up to 2% of population cohorts^27,28,32–34^.

However, the feasibility and yield of recalling carriers of psychiatric risk variants—such as NDD CNVs—from diverse, healthcare-system biobanks remain poorly characterized. Participation may be influenced by research interest and perceived relevance, logistical barriers, healthcare engagement, and sociodemographic factors, with potential implications for selection bias in RbG studies^35–38^. A previous study within the Geisinger *MyCode* Community Health Initiative identified carriers of 31 neuropsychiatric CNVs and reported mostly positive reactions to genetic disclosure among 141 carriers of nine CNVs^33^. However, the recontacted cohort was predominantly European, derived from a relatively contained healthcare system, and did not include cognitive phenotyping. In contrast, a recent analysis of the Bio*Me* biobank —a multi-ancestry biobank within the Mount Sinai healthcare system—reported a 2.5% prevalence of NDD CNVs among approximately 25,000 adults^39^. Enrichments were observed for congenital disorders and major depressive disorder, as well as for several medical conditions, including hypertension and obesity; however, that report was limited to EHR outcomes^39^.

We therefore conducted a proof-of-concept RbG study in Bio*Me*, recruiting biobank participants who harbor NDD CNVs, as well as two comparator groups without NDD CNVs: individuals with schizophrenia and neurotypical controls. In this context, we define ‘benchmarks’ as quantitative indicators of recruitment, retention, factors influencing participation, and phenotypic yield beyond EHR data. This study aimed to (i) evaluate the implementation of RbG for psychiatric risk variants in a diverse healthcare-system biobank, (ii) identify empirical benchmarks for recruitment, retention, and phenotyping yield, and (iii) explore clinical and cognitive phenotypes beyond EHR data.

## Results

### Recall by genotype, response/non-response

**Fig. 1:**
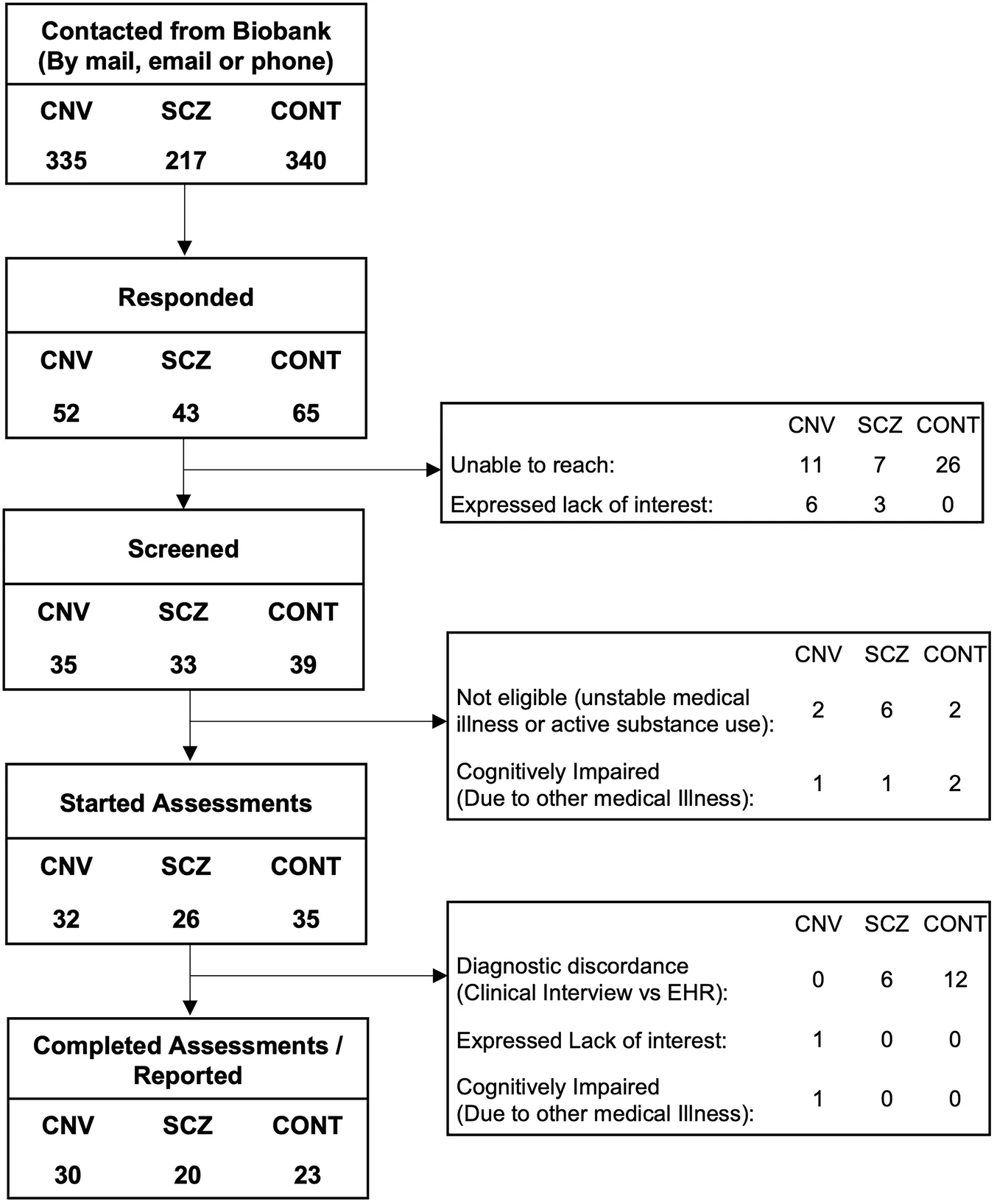
Study recruitment. Flow diagram showing recontact of 892 Bio*Me* biobank participants across three comparator groups—neurodevelopmental CNV carriers, individuals with schizophrenia without NDD CNVs, and controls without NDD CNVs—from initial contact through completion of study assessments.

Within Bio*Me*, all eligible NDD CNV carriers (*n* = 335) and all eligible individuals with schizophrenia without NDD CNVs (*n* = 217) were recontacted, whereas a subset of eligible neurotypical controls without NDD CNVs was selected for recontact (*n* = 340) to achieve comparable group sizes and demographic distributions (age, sex, and ancestry) (Table 1 and Fig. 1). The initial response rate—defined as participant reply to outreach via mail, email, or telephone—was 18%, comprised of 15% of NDD CNV carriers, 20% of individuals with schizophrenia without NDD CNVs, and 19% of neurotypical controls without NDD CNVs. Overall, 12% of recontacted participants were screened, and 10% initiated the study (Fig. 1). Attrition from initial response to study participation reflected participants who became unreachable or subsequently lacked interest (6%), self-reported alcohol or substance use (1%), or medical illness confounding cognition (1%). Among those who initiated assessments, an additional 2% of enrolled participants began the study but were excluded due to diagnostic discordance between EHR and study assessment. No significant differences were observed between the comparator groups at any stage of recruitment: responded (χ² = 2.17, *p* = 0.34), screened (χ² = 2.97, *p* = 0.23), initiated assessments (χ² = 0.84, *p* = 0.66), or completed assessments (χ² = 1.49, *p* = 0.48).

**Table 1:** Study sample

(A) Demographic characteristics of the three study groups who were recontacted and completed assessments												
	BIOBANK	BIOBANK	RECONTACTED			COMPLETED STUDY				COMPLETED vs RECONTACTED, STATISTIC		
	Total	NDD CNV	NDD CNV	SCZ	CONT	NDD CNV	SCZ	CONT	GROUP DIFFERENCE	NDD CNV	SCZ	CONT
N	24,877	627	335	217	340	30	20	23				
Mean age, Biobank enrollment (SD)	50.5 (17.3)	50.4 (17.0)	39.0 (10.8)	45.4 (9.9)	41.9 (10.7)	37.3 (9.2)	41 (9.3)	39.9 (9.4)		0.87 (0.39)	2.03 (0.05)	0.85 (0.40)
Mean age, study (SD)						47.3 [9.5]	50.2 [10.1]	49.5 [11.2]	0.55 (0.58)			
Female (%)	14,586 (59%)	389 (62%)	219 (65%)	97 (45%)	209 (61%)	26 (87%)	8 (40%)	14 (61%)	11.96 (0.003)	5.9 (0.02)	0.1 (0.78)	0.0 (0.99)
Ancestry									4.98 (0.55)	1.4 (0.70)	2.1 (0.55)	2.8 (0.43)
African	5965	168	95	101	111	10 (33%)	9 (45%)	8 (35%)	0.77 (0.68)			
European	7892	208	102	17	52	10 (33%)	2 (10%)	7 (30%)	3.73 (0.16)			
Hispanic	8536	200	109	87	151	9 (30%)	9 (45%)	7 (30%)	1.42 (0.49)			
Other	2484	51	32	12	26	1 (3%)	0 (0)	1 (4%)				

(B) Copy-number-variant (CNV) loci of the 30 NDD CNV carriers who completed assessments		
NDD CNV	hg19 coordinates	Completed study (*n* = 30)
TAR_dup	chr1:145,394,955-145,807,817	3
1q21.1del^**^	chr1:146,527,987-147,394,444	1
1q21.1dup	chr1:146,527,987-147,394,444	1
2q13del(NPHP1)	chr2:110,862,716-110,983,948	2
2q13dup(NPHP1)	chr2:110,862,716-110,983,948	3
15q11.2del	chr15:22,805,313-23,094,530	5
15q11.2dup	chr15:22,805,313-23,094,530	4
15q13.3dup(CHRNA7)	chr15:32,017,070-32,453,068	2
16p13.11del	chr16:15,511,655-16,293,689	2
16p13.11dup	chr16:15,511,655-16,293,689	2
16p11.2distal_del^**^	chr16:28,823,196-29,046,783	1
16p11.2del	chr16:29,650,840-30,200,773	1
17p12del	chr17:14,141,387-15,426,961	1
22q11.2del^**^	chr22:19,037,332-21,466,726	1
22q11.2dup	chr22:19,037,332-21,466,726	1

*SCZ* schizophrenia without NDD CNVs, *CONT* neurotypical controls without NDD CNVs. For between-group comparisons, test statistics are indicated with corresponding *p*-values in parentheses. Chi-square tests were used for categorical variables of sex and ancestry. Ancestry is as per self-report.

Thirty NDD CNV carriers completed the study. Cognitive analyses were restricted to 22 carriers to match previously reported UK Biobank analyses, excluding four loci (15q13.3 [CHRNA7] duplication, 17p12 deletion, and 2q13 [NPHP1] deletion/duplication).

^**^NDD CNV loci previously reported as significantly associated with schizophrenia.

### Study sample, demographics, and participant characteristics

Overall, 73 participants (8% of those recontacted) completed assessments: 30 NDD CNV carriers, 20 individuals with schizophrenia without NDD CNVs, and 23 neurotypical controls without NDD CNVs (Table 1). The mean age was 48.8 ± 10.2 years, 66% were female, and participants were of diverse self-identified ancestry, 37% African, 34% Hispanic, and 26% European ancestry. Age and ancestry did not differ significantly between groups. NDD CNV carriers were predominantly female (87%), whereas the schizophrenia group was mostly male (55%) (χ² = 12.0, *p* = 0.003). Among the NDD CNV group, female sex was associated with higher participation (χ² = 5.9, *p* = 0.02), and among individuals with schizophrenia, older age was associated with higher participation (*t* = 2.03, *p* = 0.05) (Supplementary Table 1). Across groups, median income as proxied by residential zip code did not differ between biobank participants who completed the study and others recontacted (*t* = 0.37, *p* = 0.71).

### Study sample and NDD CNV status

Among 30 NDD CNV carriers, 16 harbored duplications and 14 harbored deletions across 15 distinct NDD CNV loci; no participants harbored multiple NDD CNVs (Table 1 and Supplementary Table 1). Some NDD CNV loci were over-represented among participants who completed assessments (e.g., TAR duplication, 15q11.2 deletion, and 16p13.11 deletion) relative to those recontacted (Supplementary Table 1). None of the participants with schizophrenia or controls harbored NDD CNVs.

### Study sample and healthcare system utilization

Overall healthcare utilization, as proxied by the number of healthcare encounters, did not differ between participants and non-participants: the study sample averaged 125 clinical encounters versus 133 for others recontacted (*t* = 0.30, *p* = 0.76). In contrast, recent engagement was associated with participation; study participants had, on average, a clinical encounter 12 weeks prior to recontact compared with 57 weeks for non-participants (*t* = 7.1, *p* = 1.0 × 10⁻¹⁰). Illness severity, as proxied by the number of inpatient admissions, did not differ between participants and non-participants. The study sample averaged 2.1 inpatient admissions versus 2.8 for others recontacted (*t* = 1.4, *p* = 0.16).

### Clinical phenotyping outcomes

MINI administration confirmed schizophrenia diagnoses and the absence of psychiatric illness in neurotypical controls (Table 2, Fig. 1). Among the 30 NDD CNV carriers, no cases of schizophrenia were identified; however, one participant had bipolar disorder with psychosis. Mood or anxiety disorders were diagnosed in 40% (*n* = 12) of NDD CNV carriers, including five with major depressive disorder and two with obsessive-compulsive disorder. Clinical findings were often discordant with or under-reported in EHRs, particularly for developmental history, which was frequently absent from EHR documentation (Table 2). For example, childhood speech delay warranting speech therapy was reported in three NDD CNV carriers, and one NDD CNV carrier reported global developmental delay, including delayed motor milestones (e.g., walking). Formally diagnosed learning disorders were reported by approximately 30% of NDD CNV carriers, and a history of special education was reported by 13%, spanning elementary school through high school, across multiple years and multiple subject areas.

**Table 2:** Prevalence of selected neuropsychiatric and neurodevelopmental features across study groups

	NDD CNV (*n* = 30)			SCZ (*n* = 20)		CONT (*n* = 23)	
CLINICAL FEATURE	*N*	NDD CNV LOCI	EHR unreported/discordant	*N*	EHR unreported/discordant	*N*	EHR unreported/discordant
MOOD/ANXIETY DISORDER	12	TAR dup, 1q21.1 del, 1q21.1 dup, 2q13 (NPHP1) dup, 2q13 (NPHP1) dup, 15q11.2 del, 15q11.2 dup, 15q13.3 dup (CHRNA7), 16p11.2 distal del, 16p13.11 del, 16p13.11 del, 16p13.11 dup	6	0	0	0	0
SEIZURE DISORDER	1	16p11.2 distal del	1	1	1	0	0
DEVELOPMENTAL DELAY (SPEECH OR MOTOR)	4	1q21.1 del, 2q13 (NPHP1), 15q11.2 dup, 16p11.2 del	1	3	1	0	0
CONGENITAL MALFORMATIONS	3	22q11.2 deletion, 2q13 (NPHP1) del, 15q11.2 del	1	0	0	0	0
LEARNING DISORDER	8	TAR dup, 1q21.1 del, 2q13 (NPHP1) dup, 15q11.2 dup, 15q11.2 dup, 16p12.2 distal del	8	0	0	0	0
HISTORY OF SPECIAL EDUCATION	4	2q13(NPHP1) dup, 15q11.2 dup, 16p11.2 del	3	10	8	1	1
EXTREME LEARNING DIFFICULTIES	4	TAR dup, 15q11.2 del, 16p13.11 del, 22q11.2 dup	4	2	2	0	0

The number of participants affected by clinical feature, as determined by study assessments is indicated. For NDD CNV carriers, corresponding unique CNV loci are indicated. The extreme learning difficulties category is without a formally diagnosed learning disorder. One control participant reported a limited history of special education, but without a diagnosed learning disorder. Discordance between EHR data and study-assessed features is noted as counts of individuals.

*del* deletion, *dup* duplication.

A previous report detailed medical findings of the Bio*Me* NDD CNV cohort based on EHR diagnostic codes^39^. The current study assessments extended those analyses and corroborated medical findings reported in other prior CNV cohorts, including obesity, hypertension, neuropathy, and congenital anomalies (Supplementary Table 2). No congenital anomalies were observed in participants with schizophrenia or controls. No participants in this study exhibited short stature.

Regarding functional status, all NDD CNV carriers and controls reported a history of employment without disability, whereas 20% of the schizophrenia group reported disability and unemployment. All NDD CNV carriers and neurotypical controls without NDD CNVs were able to live independently, while 20% of the schizophrenia group lived in supervised residences or were unable to live independently.

### Cognitive phenotyping outcomes

**Fig. 2:**
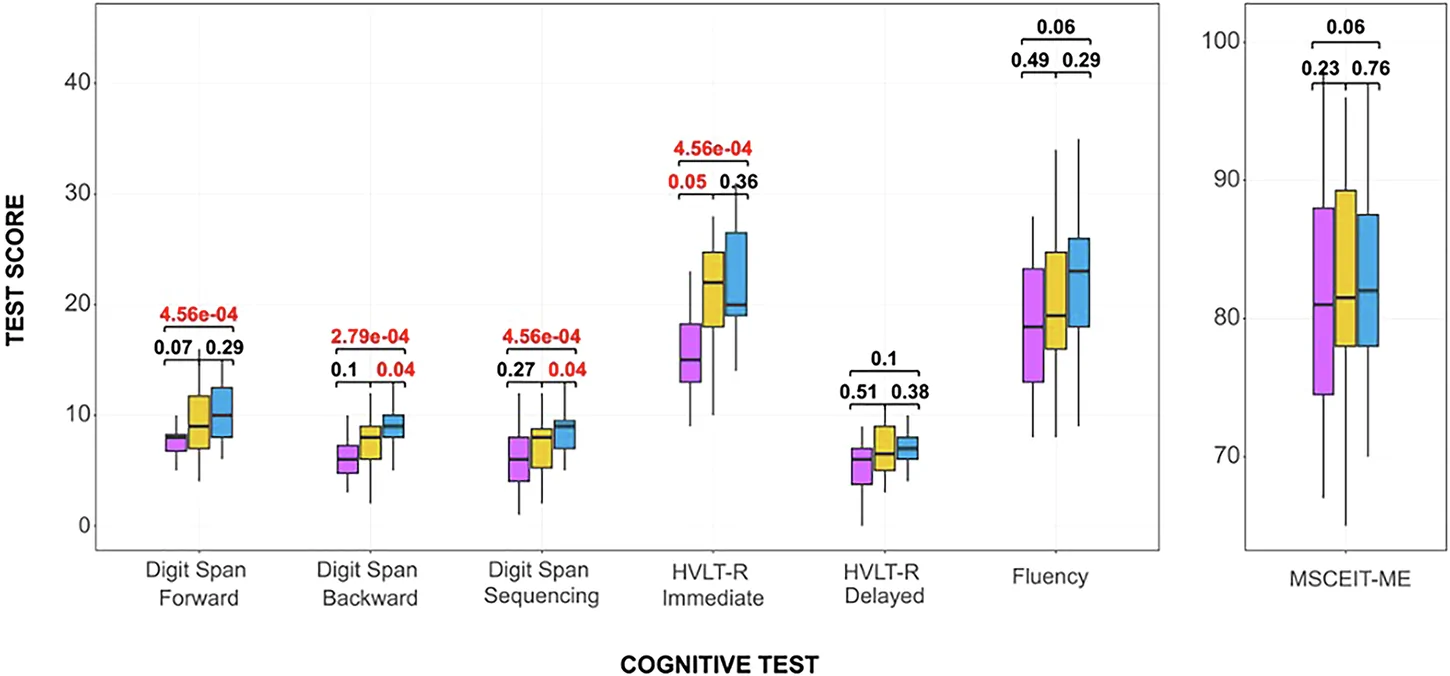
Cognitive performance by group. Boxplots showing cognitive test scores across seven cognitive domains for 22 NDD CNV carriers, 20 individuals with schizophrenia, and 23 controls. Each score was modeled by group, adjusting for age, sex, and ancestry. FDR-adjusted *p*-values are shown; red indicates significance at FDR ≤ 0.05. The x-axis represents cognitive tests, and the y-axis shows raw cognitive test scores. Each score was regressed on group status, adjusting for age, sex, and ancestry. FDR-adjusted values are shown for each test. Red indicates statistical significance at an FDR threshold of 0.05.

For cognitive analyses, the NDD CNV group was restricted to loci reported in previous UK Biobank analyses to facilitate cross-study comparability, excluding the common 15q13.3 (*CHRNA7*) duplication, 17p12 deletion, and 2q13 (*NPHP1*) deletion/duplication, yielding 22 carriers across 11 unique loci^27,28^. The 22 NDD CNV carriers performed worse than controls on Digit Span Backward (β = −1.76, FDR = 0.04) and Digit Span Sequencing (β = −2.01, FDR = 0.04); however, they outperformed the schizophrenia group on HVLT-R Immediate Recall (β = 4.5, FDR = 0.05) (Fig. 2 and Supplementary Table 4).

Ancestry and sex were both associated with variation in social cognition (ancestry: *r*² = 0.36, *p* = 6.5 × 10⁻⁷; sex: *r*² = 0.12, *p* = 0.003), and sex was additionally associated with verbal learning (*r*² = 0.11, *p* = 0.004). Consistent with locus-level heterogeneity, substantial inter-individual variation was observed across CNVs (Supplementary Table 4 and Supplementary Fig. 2). For example, the 22q11.2 deletion carrier was among the lowest-performing individuals, whereas the 1q21.1 duplication carrier was among the highest-performing across cognitive tests.

## Discussion

As clinical biobanks expand, RbG enables direct phenotyping to refine genotype–phenotype relationships, generate more precise mechanistic hypotheses, and inform downstream translational research. To our knowledge, this is the first RbG investigation of NDD CNV carriers in a multi-ancestry healthcare system—and the first recall of individuals with idiopathic schizophrenia as comparators—from a healthcare-system-based biobank. For RbG studies, ancestrally diverse representation will be essential to ensure the validity and population generalizability of genomic findings and to mitigate ancestry-related biases that can influence variant interpretation and risk estimation. This study provides initial benchmarks for RbG implementation and phenotypic yield in diverse healthcare-system biobanks.

In this study, 892 adults were recalled from Bio*Me* for participation. Overall, 18% responded to initial recruitment, 12% were screened, 10% began the study, and 8% completed study assessments. While the overall recall rate was lower than some previously reported biobank RbG studies, the resulting sample size was comparable to earlier RbG reports, most of which did not target psychiatric risk variants or include individuals with psychiatric illness (Supplementary Table 5)^10,40–49^. The relatively low completion rate raises concern for potential selection bias. Consistent with this, participation was associated with recent engagement with the healthcare system and varied modestly by sex and age, with comparable rates across ancestry and comparator groups, including controls. Other potential factors—such as perceived personal relevance or broader sociocultural and socioeconomic factors—warrant further evaluation in future studies to inform strategies for optimizing RbG study design and scalability. ZIP code–level proxy measures of socioeconomic status, as used in the current analysis, may not accurately reflect individual-level socioeconomic status and can obscure within-area heterogeneity, particularly among participants receiving disability benefits or residing in supported housing. Furthermore, EHRs may largely omit critical socioeconomic factors pertaining to health^50^. Nearly half of the control participants who initially expressed interest did not proceed to study enrollment, perhaps reflecting logistical barriers (e.g., competing work or caregiving demands) or participant burden. Future studies may evaluate strategies to enhance participation, including the use of appropriate incentives, flexible scheduling and follow-up strategies, participant-centered engagement approaches, and interventions that reduce logistical and access-related barriers.

Earlier work from the Bio*Me* biobank examined participant recontact in the context of returning clinically actionable genomic results to 74 participants for hereditary breast and ovarian cancer syndrome, Lynch syndrome, and familial hypercholesterolemia, after obtaining consent for confirmatory clinical testing^51^. The genomic screening initiative reported high participant interest but also noted challenges in re-establishing contact with some previously enrolled individuals. The current study extends genotype-based recontact from Bio*Me* into deep clinical and cognitive phenotyping for NDD CNVs—a use of biobank infrastructure not previously evaluated.

Notably, the current study demonstrated that individuals with schizophrenia, including those with functional impairment, were recalled at rates broadly comparable to controls and other groups. In addition, differences between study assessments and EHR documentation highlighted potential limitations of EHR-derived phenotypes for biobank research, particularly for childhood developmental histories that may be absent in adult EHRs. In the present study, EHRs lacked documentation of developmental histories, whereas study assessments identified developmental features among NDD CNV carriers—13% with speech or motor developmental delays, 27% with learning disorders, and 13% with a history of special education. These findings suggest that biobank-based recontact can yield clinically relevant developmental and cognitive phenotypes absent from EHRs, thereby complementing EHR-derived analyses and providing data that may support genotype–phenotype interpretation in future research, even in the absence of variant-level interpretation in the current analysis. Clinical and cognitive findings among NDD CNV carriers should be interpreted in the context of modest sample size, but were broadly consistent with prior, larger studies, providing preliminary support for external validity. Prior reports were derived from predominantly European-ancestry cohorts, and variable expressivity of NDD CNVs is known^19,27,33,52,53^. The current study also assessed social cognition, a domain not included in several prior population-based cohort studies.

Genetic results were not disclosed to participants, consistent with the IRB-approved, research-only design, since initial genotyping was not performed in a CLIA-certified laboratory, precluding a clinical return of results. This approach contrasts with the recall report from Geisinger *MyCode*, which returned results for CNVs at nine loci^33^. For the current study, consent and recontact procedures emphasized the study’s observational nature and absence of direct clinical feedback. Participants were informed during recruitment that individual genetic results would not be returned, which may have influenced participation decisions and should be considered in the design of future RbG studies. Future studies may further address ethical, legal, and social (ELSI) challenges of biobank RbG, especially to guide decision-making around disclosure of genetic variants, including variants that are not ACMG secondary findings or immediately clinically actionable^36–38,54,55^. Evolving consent models, including broader or dynamic consent frameworks, may also be important considerations for future biobank-based RbG studies.

Past RbG reports in psychiatry have been limited. The previously published recall of neuropsychiatric CNV carriers differed from the present study in that it was conducted within a relatively contained healthcare system, recruited mostly individuals of European ancestry, did not include cognitive assessments, and offered return of results^33^. Another RbG study recalled 197 healthy individuals at extremes of low or high schizophrenia polygenic risk score (PRS) from a population cohort, the Avon Longitudinal Study of Parents and Children (ALSPAC), for deep phenotyping, including behavioral assessments and structural and functional imaging^56^. The study identified an effect of schizophrenia PRS on psychotic experiences and blood oxygen level-dependent signal during reward processing in the ventral striatum and a broader reward-related network, but no effect on whole-brain measures or brain morphometry. Another RbG study from ALSPAC recalled 24 individuals who harbored a schizophrenia common risk variant in *ZNF804A* for deep phenotyping and queried the effect of the risk variant on sleep architecture and memory^57^. Neither of the RbG studies from ALSPAC returned genetic results.

Beyond psychiatry, the few published biobank RbG studies have mostly focused on genetic variants for cardiometabolic traits or cancer (Supplementary Table 5)^9,11^. The sample sizes of previous biobank RbG reports have varied by biobank, genomic variant frequency, target population, recall method, and study goals, with some studies extending recruitment to family cascade screening^10,40–49^. The study completion rate of 8% is lower than some past biobank RbG reports, underscoring the challenges of RbG studies; however, many did not report recall rates, precluding direct comparison. Most past RbG studies were descriptive, deploying phenotyping methods for recalled participants, some including remote assessments. One RbG study incorporated Mendelian Randomization analysis to enhance causal inference^43^. A recent review of biobank RbG studies emphasized that additional RbG studies are “urgently needed” to optimize and leverage the study design, especially for follow-up longitudinal research^9,11,48^.

This study had several limitations. The modest sample size, particularly for NDD CNV carriers, limited statistical power and precluded locus-specific or subgroup analyses. Accordingly, CNV analyses were pooled across loci to maximize statistical power. The study was not powered to draw definitive conclusions regarding genotype–phenotype associations. The remote administration of assessments, though validated, could have influenced performance; however, it also informs scalability and inclusion of geographically distant participants. The clinical assessments were not designed to evaluate NDD CNV pathogenicity, which has been addressed in prior genetic studies. Sex and ancestry were included as covariates in pooled analyses, but stratified analyses were not feasible given the sample size. Some clinical assessments relied on participant self-report and may be subject to recall bias. Although the study excluded medical conditions known to contribute to cognitive impairment, other potential confounders, such as medication burden, were not controlled. Analyses focused on a subset of recurrent NDD CNV loci and did not incorporate genome-wide CNV burden or other rare non-recurrent CNVs. NDD CNVs were selected based on prior biobank analyses, with loci of variable effect sizes that may have attenuated group-level findings. Low-functioning NDD CNV carriers may have been underrepresented due to reduced likelihood of study participation, potentially biasing results. Accordingly, recruitment yield estimates may represent conservative benchmarks for lower-functioning carriers. The female overrepresentation in the NDD CNV group may limit generalizability; future studies should aim for balanced sex representation. These analyses provide some initial quantitative benchmarks—including response rates, participation patterns, and phenotyping outcomes—that can inform future RbG design, scalability, and implementation.

This study establishes a proof-of-concept for RbG of a class of psychiatric risk variants from a multi-ancestry, healthcare-system biobank. The ability to assemble genotypically defined cohorts for follow-up observational or interventional research—both cross-sectional and longitudinal—represents an opportunity for advancing genomic and neuropsychiatric research. Extending RbG to additional clinical biobanks in healthcare systems, and targeting distinct psychiatric genetic risk variants, may offer an actionable framework to translate disease-relevant genetic findings into clinical insights. Future research evaluating the scalability and generalizability of RbG approaches will be essential to refine genotype–phenotype relationships and advance precision psychiatry.

## Methods

### Ethics approval

The Bio*Me* biobank is an EHR-linked resource of genotyped individuals of diverse ancestry, recruited across the Mount Sinai Health System (New York, NY) since 2007 and approved by the Institutional Review Board (IRB) at the Icahn School of Medicine at Mount Sinai^39,58^. As previously described, biobank participants were recruited across the healthcare system, primarily from outpatient medicine clinics, with some recruitment from outpatient psychiatry clinics^39^. For the present study, the IRB of the Icahn School of Medicine at Mount Sinai approved BioMe data access, participant recontact, informed consent, and study assessments (STUDY-19-00095). All procedures involving human participants were conducted in accordance with the ethical standards of the institutional research committee and with the Declaration of Helsinki. All participants who enrolled in the study provided informed consent prior to participation. The IRB also approved a waiver of informed consent for retrospective chart review to facilitate identification of eligible participants for recontact. Recontact of participants did not include return of genetic results, consistent with the use of non-CLIA-certified research data. Participants were informed during recruitment, and all recruitment materials emphasized that participation would not result in the return of individual genetic results.

### Recruitment

Within Bio*Me*, we identified three groups of adults, ages 18-65 years, for recontact: (i) NDD CNV carriers, harboring at least one NDD CNV; (ii) individuals with schizophrenia without NDD CNVs, defined by ≥ 2 International Classification of Diseases (ICD-10) diagnoses for schizophrenia; and (iii) neurotypical controls without NDD CNVs, defined as having no ICD-10 neuropsychiatric diagnoses. The age range was selected to focus on adults while minimizing confounding from age-related cognitive decline. NDD CNVs were defined using a previously published list of 92 recurrent CNVs across 47 genomic regions—comprising reciprocal deletions and duplications—previously reported to be associated with neurodevelopmental and psychiatric phenotypes^27,28,32,39^.

Bio*Me* participants identified for recall were contacted by mail and email (up to two attempts each), followed by telephone outreach with multiple attempts on multiple days and times. Phone calls were limited to weekdays between 8:00 AM and 6:00 PM. Recontact was paused if a participant expressed a lack of interest or after six unsuccessful telephone attempts.

Respondents were excluded for unstable or severe medical illness, current substance or alcohol use disorder, or other conditions expected to affect cognition. A chart review was conducted by a board-certified psychiatrist (RB) to confirm group assignment (schizophrenia or neurotypical control) and overall medical history. EHRs were reviewed from January 1, 2005, through November 12, 2020, including inpatient and outpatient encounter notes, procedure notes, and medication prescription records.

### Clinical and cognitive assessments

Assessments were performed remotely in two 30-minute sessions scheduled at participant preference and completed at two non-contiguous timepoints (Supplementary Fig. 1). The clinical assessment consisted of a validated, structured diagnostic interview, the Mini International Neuropsychiatric Interview (MINI v7.0.2), compatible with DSM-5 and ICD-10 criteria^59^. In addition, a semi-structured clinical interview was conducted to obtain a medical and psychiatric history (including medication use and prior hospitalizations), as well as a retrospective developmental history, and social and occupational histories to assess functional status.

Cognitive assessments were administered by trained staff (NZ) using standardized instructions and scoring. To prioritize assessment of cognitive domains most relevant to study objectives, we selected seven subtests that assess domains implicated in NDD CNVs and validated in both neuropsychiatric and neurotypical populations: digit span (forward, backward, and sequencing) to assess attention and executive function; category fluency to assess processing speed and semantic memory; the Mayer–Salovey–Caruso emotional intelligence test—Managing Emotions branch (MSCEIT-ME) to assess social cognition; and the Hopkins Verbal Learning Test–Revised (HVLT-R) to assess immediate and delayed verbal memory^60,61^. The immediate recall score represented the total number of words recalled across three learning trials, consistent with the MATRICS Consensus Cognitive Battery (MCCB) protocol. The MSCEIT was scored using the standardized MSCEIT software^62^.

### Remote administration

Evidence supports the validity of remote administration of the clinical and cognitive scales used in this study, including telephone-based assessments among individuals with schizophrenia and cognitive impairment^63–68^.

### EHR visit density

Visit density was calculated as the total number of completed clinical encounters with a healthcare provider from January 1, 2005, through November 12, 2020 (visit-level EHR data), with inpatient and outpatient encounters each counted as one visit.

### Median income estimates

Residential zip codes of participants were linked to area-level median household income using publicly available data from the U.S. Census Bureau American Community Survey (ACS)^69^.

### Statistical analyses

Cognitive raw scores were modeled using linear regression to compare groups, with age, sex, and self-reported ancestry category included as covariates. Self-reported ancestry categories were obtained from biobank enrollment questionnaires and used for descriptive reporting and adjustment in regression analyses, but not treated as proxies for genetic ancestry^39^. All analyses were performed in R 4.0.4. Statistical significance was evaluated using the Benjamini–Hochberg false discovery rate (FDR) procedure.

## Supplementary information

Supplementary information

## Data Availability

Only de-identified results are contained in the publication. The datasets analyzed during the current study are not publicly available since they contain identifiable information from BioMe participants.

## Code availability

Analyses were performed using standard software. No custom software was developed.
