# Supplementary material for: Recall-by-genotype of neurodevelopmental disorder copy number variants in a multi-ancestry, healthcare-system biobank
Source: NPJ Genom Med. 2026 Jul 16;11:61. doi: 10.1038/s41525-026-00597-6 (PMC13745509; doi:10.1038/s41525-026-00597-6)
Supplement: Supplementary file 1 [file 41525_2026_597_MOESM1_ESM.pdf]

Supplementary Table 1: NDD CNV Loci, Prevalence. Counts of NDD CNV carriers, by locus, with chromosomal coordinates indicated.

NDD CNV	hg19 coordinates	Genes (n)	BioMe (n=627)	Recontacted (n=335)	Completed Study (n=30)	NDD CNV Prevalence: BioMe Total vs Study (p-value)
TAR_del	chr1:145,394,955-145,807,817	17	5	2		
TAR_dup	chr1:145,394,955-145,807,817	17	18	8	3	2.29E-05
1q21.1del**	chr1:146,527,987-147,394,444	9	8	5	1	0.27
1q21.1dup	chr1:146,527,987-147,394,444	9	3	2	1	0.05
NRXN1_DEL**	chr2:50145643-51259674	1	3	1		
2q11.2del	chr2:96,742,409-97,677,516	22	2	1		
2q11.2dup	chr2:96,742,409-97,677,516	22	1	1		
2q13del(NPHP1)	chr2:110,862,716-110,983,948	3	81	38	2	0.70
2q13dup(NPHP1)	chr2:110,862,716-110,983,948	3	62	32	3	0.08
2q13del	chr2:111,394,040-112,012,649	3	3	3		
2q13dup	chr2:111,394,040-112,012,649	3	8	7		
2q21.1del	chr2:131,481,308-131,930,677	5	9	6		
2q21.1dup	chr2:131,481,308-131,930,677	5	5	3		
3q29del**	chr3:195,720,167-197,354,826	28	1	1		
3q29dup	chr3:195,720,167-197,354,826	28	0	0		
Sotos_5q35del	chr5:175,720,924-177,052,594	39	0	0		
5q35dup	chr5:175,720,924-177,052,594	39	0	0		
7q11.23_del	chr7:72,744,915-74,142,892	26	1	0		
7q11.23_dup	chr7:72,744,915-74,142,892	26	0	0		
7q11.23dup_distal	chr7:75,138,294-76,064,412	16	0	0		
8p23.1del	chr8:8,098,990-11,872,558	35	0	0		
8p23.1dup	chr8:8,098,990-11,872,558	35	0	0		
10q11.21q11.23del	chr10:49,390,199-51,058,796	19	1	0		
10q11.21q11.23dup	chr10:49,390,199-51,058,796	19	2	0		
10q23del	chr10:82,045,472-88,931,651	29	0	0		
10q23dup	chr10:82,045,472-88,931,651	29	1	0		
13q12del(CRYL1)	chr13:20,977,806-21,100,012	2	8	4		
13q12dup(CRYL1)	chr13:20,977,806-21,100,012	2	0	0		
13q12.12del	chr13:23,555,358-24,884,622	10	4	3		
13q12.12dup	chr13:23,555,358-24,884,622	10	9	3		
15q11.2del	chr15:22,805,313-23,094,530	5	60	27	5	8.01E-05
15q11.2dup	chr15:22,805,313-23,094,530	5	163	89	4	0.39
PW/AS_15q11.2q13.1 BP1-3_del	chr15:22,805,313-28390339	116	0	0		
PW/AS_15q11.2q13.1 BP1-3_dup	chr15:22,805,313-28390339	116	0	0		
15q11q13del_BP3-BP4(APBA2_TJP)	chr15:29,161,368-30,375,967	4	1	1		
15q11q13dup_BP3-BP4(APBA2_TJP)	chr15:29,161,368-30,375,967	4	3	1		
15q11q13dup_BP3-BP5	chr15:29,161,368-32462776	17	0	0		
15q13.3del	chr15:31,080,645-32,462,776	8	5	0		
15q13.3dup	chr15:31,080,645-32,462,776	8	5	2		
15q13.3del(CHRNA7)	chr15:32,017,070-32,453,068	1	5	2		
15q13.3dup(CHRNA7)	chr15:32,017,070-32,453,068	1	42	19	2	0.23
15q24del	chr15:72900171-78151253	77	0	0		
15q24dup	chr15:72900171-78151253	77	0	0		
16p13.11del	chr16:15,511,655-16,293,689	7	13	4	2	3.35E-03
16p13.11dup	chr16:15,511,655-16,293,689	7	39	19	2	0.19
16p12.1del	chr16:21,950,135-22,431,889	8	2	0		
16p12.1dup	chr16:21,950,135-22,431,889	8	14	7		
16p11.2distal_del**	chr16:28,823,196-29,046,783	11	5	4	1	0.13
16p11.2distal_dup	chr16:28,823,196-29,046,783	11	4	3		
16p11.2del	chr16:29,650,840-30,200,773	30	15	12	1	0.53
16p11.2dup**	chr16:29,650,840-30,200,773	30	4	4		
17p12del	chr17:14,141,387-15,426,961	8	6	5	1	0.18
17p12dup	chr17:14,141,387-15,426,961	8	10	7		
Smith Magenis Syndrome_17p11.2del	chr17:16,812,771-20,211,017	59	0	0		
Potocki-Lupski syndrome_17p11.2dup	chr17:16,812,771-20,211,017	59	0	0		
17q11.2del(NF1)	chr17:29,107,491-30,265,075	19	0	0		
17q11.2dup(NF1)	chr17:29,107,491-30,265,075	19	0	0		
17q12del	chr17:34,815,904-36,217,432	17	4	2		
17q12dup	chr17:34,815,904-36,217,432	17	4	2		
17q21.31del	chr17:43,705,356-44,164,691	10	0	0		
22q11.2del**	chr22:19,037,332-21,466,726	61	1	1	1	3.46E-03
22q11.2dup	chr22:19,037,332-21,466,726	61	7	4	1	0.22
22q11.2distal_del	chr22:21,920,127-23,653,646	26	0	0		
22q11.2distal_dup	chr22:21,920,127-23,653,646	26	0	0		

***=NDD CNV loci previously reported to be significantly associated with schizophrenia by GWAS (Marshall CR, et al. Contribution of copy number variants to schizophrenia from a genome-wide study of 41,321 subjects. Nat Genet. 2017;49(1):27-35).*
p-value is for a Test of Equal Proportions

Supplementary Table 2: Selected Medical Features, by NDD CNV locus. Overlap of medical features identified during current assessment with conditions previously reported in other larger-scale cohorts.

CNV locus	Location (hg19)	BioMe CNV Sample (n)	Medical Phenotypes overlapping UK Biobank or Previous Reports*
TAR_dup	chr1:145,39-145,81	3	Obesity
1q21.1del	chr1:146,53-147,39	1	
1q21.1dup	chr1:146,53-147,39	1	Gastric Ulcers
2q13del (*NPHP1*)	chr2:110,86-110,98	2	Congenital Cardiac Anomaly
2q13dup (*NPHP1*)	chr2:110,86-110,98	3	
15q11.2del	chr15:22,81-23,09	5	Congenital renal anomaly
15q11.2dup	chr15:22,81-23,09	4	
15q13.3dup (*CHRNA7*)	chr15:32,02-32,45	2	
16p13.11del	chr16:15,51-16,29	2	
16p13.11dup	chr16:15,51-16,29	2	Hypertension
16p11.2distal_del	chr16:28,82-29,05	1	Obesity
16p11.2del	chr16:29,65-30,20	1	Obesity, Hypertension
17p12del(HNPP)	chr17:14,14-15,43	1	Neuropathy
22q11.2del	chr22:19,04-21,47	1	Congenital Cardiac Anomaly, Thrombytopenia
22q11.2dup	chr22:19,04-21,47	1	

**Complete medical histories of NDD CNV carriers are not indicated, but rather only conditions that overlap previous reports in other cohorts, as summarized in Crawford K. et al. Medical consequences of pathogenic CNVs in adults: analysis of the UK Biobank. J Med Genet. 2019;56(3):131-8 Medical phenotypes during current study were determined by a consensus of chart review, clinical interview and patient self-report*

For statistical associations of NDD CNVs with ICD codes, see Birnbaum et al. Clinical Characterization of Copy Number Variants Associated With Neurodevelopmental Disorders in a Large-scale Multiancestry Biobank. JAMA Psychiatry. 2022;79(3):250-9.

Supplementary Table 3: Cognitive Effects of NDD CNVs: Results of linear regression analyses of seven cognitive tests, compared by group status.

NDD CNV subset (n=22) compared to schizophrenia (n=20) and controls (n=23)

Cogntive Test	β		t-value		p-value		FDR	
	Controls	Schizophrenia	Controls	Schizophrenia	Controls	Schizophrenia	Controls	Schizophrenia
DigitSpan_Forward	1.10	-2.00	1.41	-2.38	0.16	0.02	0.29	0.07
DigitSpan_Backward	1.76	-1.54	2.57	-2.08	0.01	0.04	**0.04**	0.10
DigitSpan_Sequencing	2.01	-1.10	2.60	-1.33	0.01	0.19	**0.04**	0.27
HVLTR_Immediate	1.69	-4.50	1.14	-2.79	0.26	0.01	0.36	**0.05**
HVLTR_Delayed	0.80	-0.57	0.99	-0.66	0.33	0.51	0.38	0.51
Fluency	2.82	-1.73	1.44	-0.82	0.16	0.42	0.29	0.49
MSCEIT-ME	0.63	-3.37	0.31	-1.53	0.76	0.13	0.76	0.23

Reference group: CNV

Schizophrenia (n=20) vs Controls (n=23)

Cogntive Test	β	t-value	p-value	FDR
DigitSpan_Forward	-3.10	-3.89	2.30E-04	**4.56E-04**
DigitSpan_Backward	-3.24	-4.40	3.99E-05	**2.79E-04**
DigitSpan_Sequencing	-3.07	-3.86	2.60E-04	**4.56E-04**
HVLTR_Immediate	-6.12	-3.93	2.02E-04	**4.56E-04**
HVLTR_Delayed	-1.33	-1.65	0.10	0.10
Fluency	-4.39	-2.07	0.04	0.06
MSCEIT-ME	-4.03	-1.97	0.05	0.06

Reference group: Controls

Notes: β coefficients from linear models adjusted for age, sex, and ancestry. p values are FDR-corrected for multiple comparisons (FDR ≤ 0.05 shown in bold).

Supplementary Table 4. Cognitive Test Performance Rank, by NDD CNV Locus. Rank of cognitive scores by NDD CNV locus.

NDD CNV Locus	Overall_Rank	Digit Span Forward	Digit Span Backward	Digit Span Sequencing	HVLT-R Immediate	HVLT-R Delayed	Fluency	MSCEIT-ME
1q21.1 dup	1	6	2	1	8	2	2	6
15q13.3 dup (CHRNA7)	2	3	1	2	10	12	1	25
22q11.2 dup	3	1	14	6	19	12	9	4
16p11.2distal del	4	11	19	6	2	12	10	8
16p13.11 dup (2)	5	19	9	19	3	1	16	6
15q13.3 dup (CHRNA7) (2)	6	17	9	12	1	12	14	10
TAR dup	7	11	14	12	14	5	4	20
2q13 del (NPHP1)	8	11	14	6	8	12	19	18
16p13.11 del (2)	9	6	19	6	4	17	12	26
2q13 dup (NPHP1) (3)	10	17	9	19	12	18	5	14
2q13 del (NPHP1) (2)	11	22	4	19	9	5	8	28
15q11.2 del (5)	12	17	19	23	8	6	7	16
17p12 del	13	17	19	19	8	5	4	25
16p13.11 dup	14	6	9	19	16	23	23	7
2q13 dup (NPHP1)	15	17	19	21	17	13	7	21
15q11.2 dup (4)	16	3	21	12	22	17	23	18
15q11.2 del (3)	17	17	9	12	14	19	28	20
15q11.2 dup (2)	18	26	21	25	19	12	14	9
15q11.2 dup (3)	20	11	14	12	22	30	25	22
16p13.11 del	20	22	27	19	24	30	11	3
15q11.2 dup	21	7	14	21	25	30	25	16
15q11.2 del	22	27	25	12	11	17	27	NA
TAR dup (2)	23	19	25	25	22	22	18	13
TAR dup (3)	24	22	25	27	16	14	18	28
15q11.2 del (4)	25	30	29	30	24	20	15	13
1q21.1 del	26	26	3	19	30	24	30	30
16p11.2 del	27	28	30	23	26	21	23	23
22q11.2 del	28	26	25	27	30	30	26	13
15q11.2 del (2)	29	26	29	30	30	30	23	NA
2q13 dup (NPHP1) (2)	30	30	27	30	30	30	30	29

Note: For ranking of Digit Span tests and HVLT-R, cognitive scores were adjusted for age, sex, race/ancestry and years of education . For ranking of Fluency and MSCEIT-ME scores were adjusted for age, sex, and years of education. (See Supplementary Methods)

A rank of '1' is highest performing and '30' is lowest (most impaired). See Supplementary Figure 1 for a plot of rankings, by NDD CNV carrier and by cognitive test

Parentheses indicate the number of carriers if more than one carrier

Supplementary Table 5. Recall-by-Genotype Studies in Biobanks (Non-Psychiatric). Summary of RbG of non-psychiatric risk variants, showing target variant, recall rates, sample sizes, and main findings.

Disease Area	Genetic Variant(s)	Biobank	Study Design	Number of Participants	Recall Rate	Main Results	Genetic Disclosure / Return-of-Results?	PubMed ID
Diabetes	IL2RA haplotypes (rs12722495, rs11594656, rs2104286)	Cambridge BioResource	Recalled individuals by haplotype to measure IL2RA surface expression on T cells; flow cytometry	~200 per haplotype group	NR	Protective haplotype had increased IL2RA expression on memory CD4+ T cells; functional link to T1D risk	No	19701192
Breast, Ovarian Cancer	BRCA1 / BRCA2 pathogenic variants	Estonian Biobank	RbG for clinical follow-up, genetic counseling,	40	55%	22 responded and received results; 8 high-risk, 5 underwent risk-reducing surgery, 10 adhered to surveillance, 10 relatives screened	Yes	33230308
Familial Hypercholesterolemia (FH)	Rare variants pathogenic for FH in LDLR / APOB / PCSK9	Estonian Biobank	RbG for clinical/lipid testing + genetic counseling	27 probands + 20 family members (Cascade screening)	70%	Reclassified 51% of the study participants from nonspecific hypercholesterolemia to having FH; 32% were unaware of harboring a high-risk disease-associated genetic variant	Yes	35928446
Wilson's Disease	ATP7B variants	Estonian Biobank	RbG for deep phenotyping (clinical assessment) and serum copper measurements	17	88%	Identified underdiagnosed WD carriers, many with early neurodegenerative signs	Yes	39674827
Body Mass Index and Cardiovascular Health	BMI GRS extremes	ALSPAC	RbG at Age 21 for top vs bottom BMI GRS percentiles for cardiovascular phenotyping	418 proposed	20%	Differences in BMI, SBP across extremes; genotype-based stratification reduced confounding	No	30524135
Smoking / Nicotine Metabolism	CHRNA5-A3-B4 genotype	ALSPAC	RbG to compare cotinine levels, smoking topography	200 proposed	NR	Test genotype-phenotype relationships in smoking	No	24451018
Adiponectin / Metabolic Traits	ADIPOQ variants	Exeter 10,000	RbG of carriers vs non-carriers for metabolic phenotyping	12	NR	Functional genomic investigations of adiponectin biology (i.e. ADIPOQ splicing)	No	26996131
Obesity	AMY1 copy number variation	DESIR	RbG to compare metabolomic signatures of high- and low-copy number carriers, Measured serum pancreatic and total amylase levels	100	NR	Identified differences in lipid metabolism between the two groups	No	35928446
Alzheimer's Disease	Alzheimer's Disease (AD) risk SNPs	Finnish National Biobank	RbG for digital cognitive and remote assessments	27	19%	Identified cognitive deficits and blood-based biomarkers distinguishing AD from mild cognitive disorder.	No	37537264
Alzheimer's Disease	AD-Polygenic Risk Score extremes	PROTECT	RbG of individuals at extremes of Alzheimer's PRS for memory and imaging assessments	16	NR	Associations with memory deficits and cingulate cortex differences	No	38087383

Notes: RbG = recall-by-genotype; NR=Not reported

<u>Supplementary Figure 1. Study Assessments, Overview</u>. Overview of clinical and cognitive assessments administered to study participants.

CLINICAL ASSESSMENT
Mini International Neuropsychiatric Interview (MINI)
Medical History
Psychiatric History (including medications and hospitalizations)
Developmental History
Social, Occupational and Educational History

COGNITIVE ASSESSMENT		
**Test**	**Domain**	**Battery**
Digit Span Forward	Attention	WAIS-IV
Digit Span Backward	Executive function, Verbal working memory	WAIS-IV
Digit Span Sequencing	Working memory maintenance	WAIS-IV
Category fluency	Processing speed	MCCB
Hopkins Verbal Learning Test - Revised (HVLT-R)	Delayed recall	MCCB
Hopkins Verbal Learning Test - Revised (HVLT-R)	Immediate recall	MCCB
MSCEIT-ME	Social Cognition, Emotional intelligence	MCCB

Notes: *MCCB = MATRICS Consensus Cognitive Battery; WAIS = Wechsler Adult Intelligence Scale*

Supplementary Figure 2. Cognitive Performance by NDD CNV locus. Plot showing the cognitive performance rank of each NDD CNV carrier across seven cognitive tests

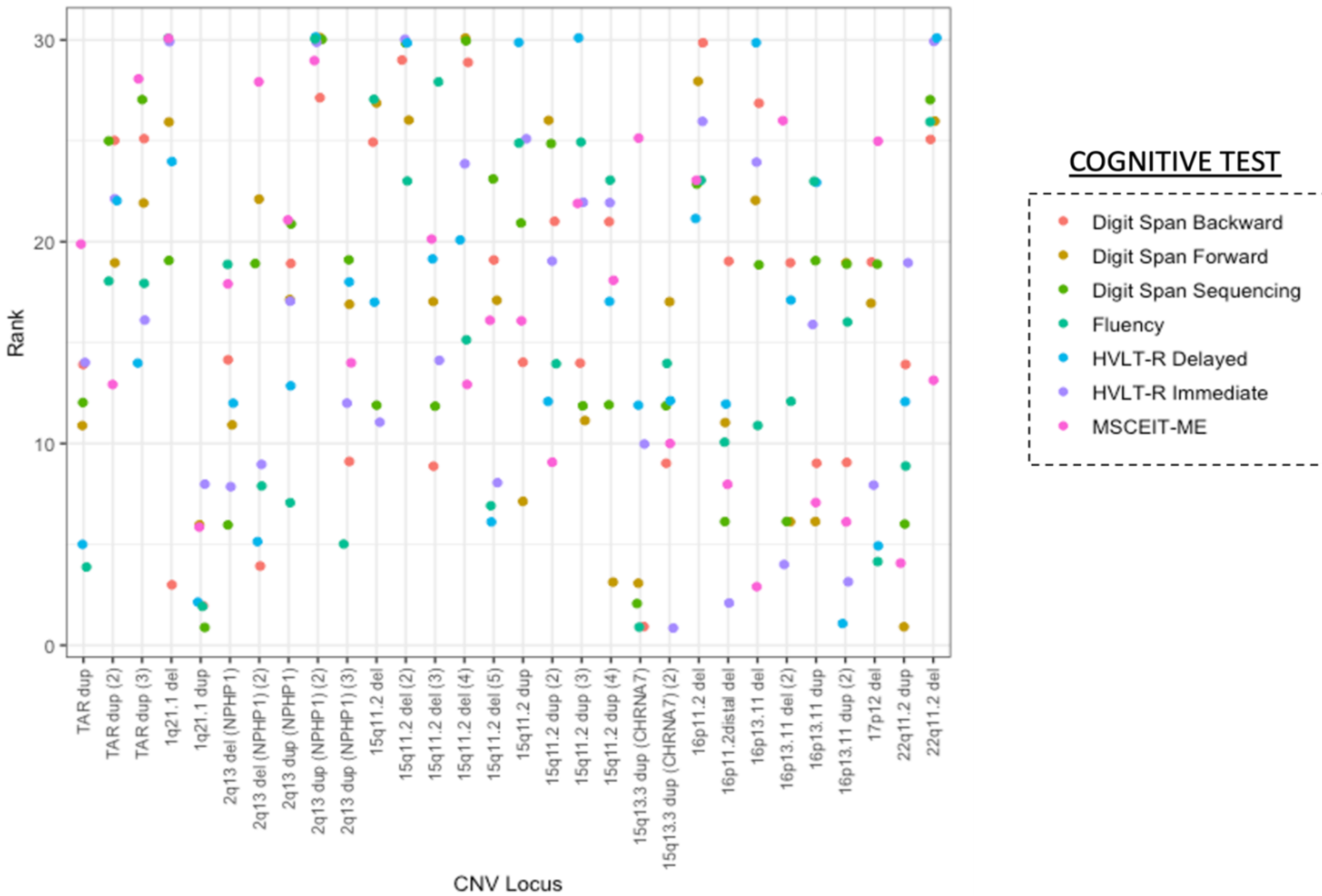

Notes: See Supplementary Table 4 for ranked loci in tabulated form.
This x-axis represents NDD CNV locus. For NDD CNV loci with more than one carrier, sequential numbers are indicated in parentheses, such as (1,2,3). The y-axis reflects rank, where 1=highest performing and 30=lowest (most impaired).
Digit Span and HVLT-R were adjusted for age, sex, race/ethnicity, and education. Scores for Fluency and MSCEIT were adjusted for age, sex, and education.
del=deletion; dup=duplication.
